# Long non-coding RNA *GAS5* and *ZFAS1* are prognostic markers involved in translation targeted by *miR-940* in prostate cancer

**DOI:** 10.18632/oncotarget.23254

**Published:** 2017-12-14

**Authors:** Xin Chen, Chao Yang, Shengli Xie, Edwin Cheung

**Affiliations:** ^1^ Guangdong Key Laboratory of IoT Information Technology, School of Automation, Guangdong University of Technology, Guangzhou, China; ^2^ Faculty of Health Sciences, University of Macau, Macau, China

**Keywords:** prognostic marker, lncRNA, co-expression, network, prostate cancer

## Abstract

Identification of prognostic biomarkers helps facilitate the prediction of patient outcomes as well as guide treatments. Accumulating evidence now suggests that long non-coding RNAs (lncRNAs) play key roles in tumor progression with diagnostic and prognostic values. However, little is known about the biological functions of lncRNAs and how they contribute to the pathogenesis of cancer. Herein, we performed weighted correlation network analysis (WGCNA) on 380 RNA-seq samples from prostate cancer patients to create networks comprising of microRNAs, lncRNAs, and protein-coding genes. Our analysis revealed expression modules that associated with pathological parameters. More importantly, we identified a gene module that is involved in protein translation and is associated with patient survival. In this gene module, we explored the regulation axis involving *GAS5*, *ZFAS1,* and *miR-940*. We show that *GAS5*, *ZFAS1,* and *miR-940* are up-regulated in tumors relative to normal prostate tissues, and high expression of either lncRNA is an indicator of poor patient outcome. Finally, we constructed a co-expression network involving *GAS5*, *ZFAS1*, and *miR-940,* as well as the targets of *miR-940*. Our results show that *GAS5* and *ZFAS1* are targeted by *miR-940* via *NAA10* and *RPL28*. Taken together, co-expression analysis of gene expression profiling from RNA-seq can accelerate the identification and functional characterization of novel prognostic markers in prostate cancer.

## INTRODUCTION

Prostate cancer (PCa) is one of the leading causes of cancer-related death for men in North America and Europe [1]. Prostate-specific antigen (PSA) analysis, biopsy, as well as the Gleason score, are diagnostic tools that have improved the diagnosis and management of PCa [2]. Among treated PCa patients, pathological parameters can predict the outcome of patients. For example, serum PSA, biopsy, and the Gleason score are well-known predictors of biological outcome following primary therapy for PCa. However, outcome prediction has shifted from pathological parameters to biological molecules. Molecular biomarkers, such as the expression of specific protein-coding and non-coding genes have now greatly improved the accuracy of outcome prediction for patients after treatment [3].

Specific functions are correlated or predictive of pathological parameters that are characteristic of tumor progression. For example, adhesion-related genes are correlated with Gleason score. Specifically, in human prostate adenocarcinomas, the down-regulation of the adhesion molecule *CD44* standard (*CD44s*) [4] and E-cadherin (*CDH1*) [5, 6] was reported to be associated with metastasis and high Gleason score. Cell cycle genes are also biomarkers that can predict the risk of clinicopathological outcomes [7] such as biochemical recurrence rate after prostatectomy therapy [8, 9]. More recently, the deregulation of ncRNAs has been associated with cell proliferation and survival of PCa [10]. Therefore, it is of great importance to determine the biological functions that are important for pathological features and to identify the corresponding novel biomarkers relevant for clinicopathological parameters, in particular, non-coding RNAs (ncRNAs), including microRNAs (miRNAs) and lncRNAs.

In this study, we examined gene modules and their corresponding biological functions that are significantly linked to clinicopathological parameters. We found potent prognostic markers, including lncRNAs that were identified based on their association with survival time. We also used WGCNA to look for gene sets with similar biological function based on the TCGA dataset for PCa. Six gene modules were identified, one of which is related to patient survival time. Enrichment analysis revealed the genes in the survival time-related module are significantly associated with the regulation of protein translation. We further identified dysregulated lncRNAs involved in protein translation with prognostic potential in PCa and dissected the roles of lncRNAs and miRNAs via target predictions and co-expression networks.

## RESULTS

### Identification of gene modules using WGCNA

**Figure 1:**
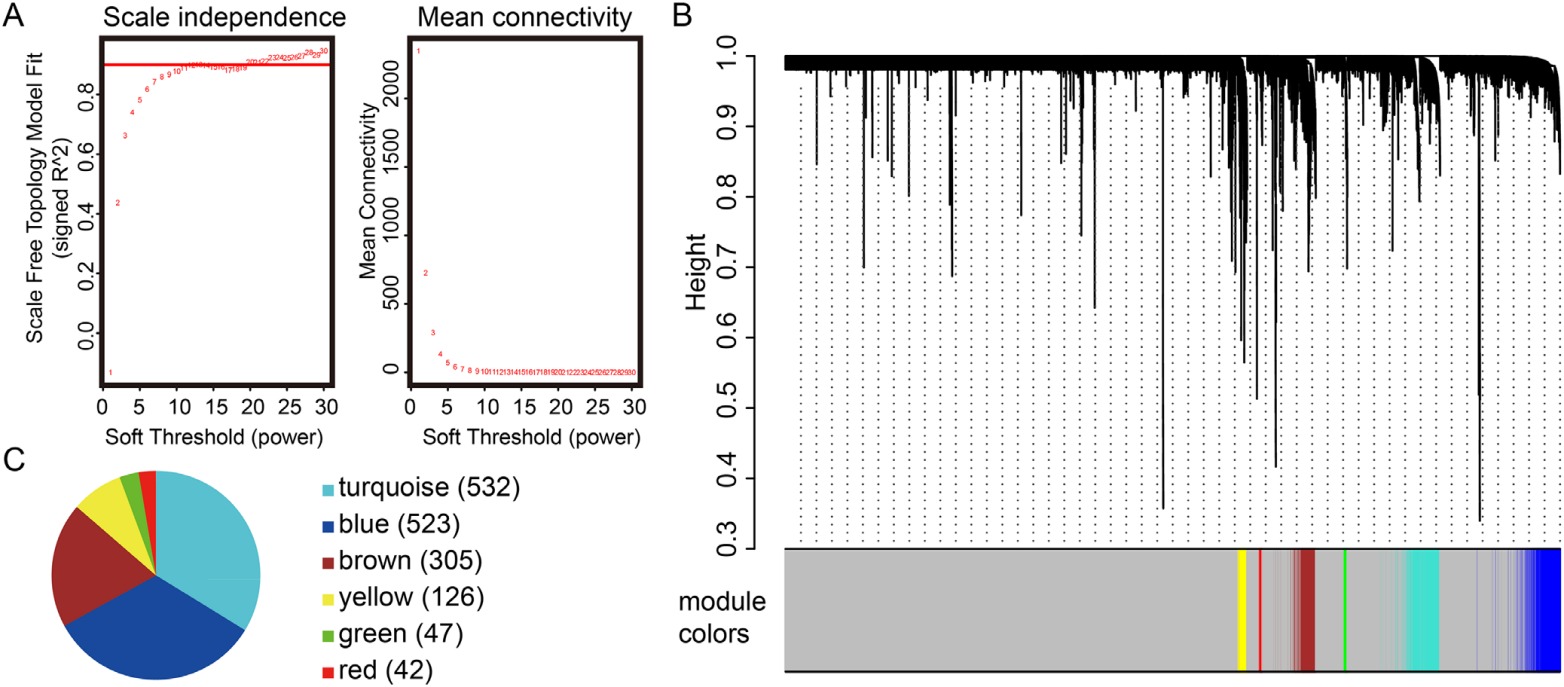
Gene modules detected using the weighted correlation network analysis (WGCNA) **(A)** Scale-free topology index and mean connectivity were used to determine the soft threshold. **(B)** Clustering dendrogram of genes. The dissimilarity of genes is based on topological overlap. The genes are assigned to different modules and are identified using different colors. **(C)** Number of genes in each module identified from WGCNA. The numbers in the bracket represent the number of genes in each module. The modules containing the most number of genes are the turquoise module, blue module and brown module.

Extending the survival time is the final goal for patients suffering from PCa. In this follow up analysis of the TCGA PRAD dataset, WGCNA was used to create co-expressed gene networks associated with survival time. Only genes with appreciable expression levels (FPKM>1) in more than half of the PCa patients were subjected to analysis. Power 22 was selected as the soft threshold to identify co-expression gene modules (for details, see the Materials and Methods section). Seven gene-network modules were identified and color-coded. Since the “grey” module is reserved for unassigned genes, we focused on the other six modules instead. As shown in Figure 1, the turquoise, blue and brown modules are the top 3 modules which contained the highest number of genes. The turquoise module contained 532 genes, while the blue and brown had 523 and 305 genes, respectively.

### Linking modules to pathological parameters

**Figure 2:**
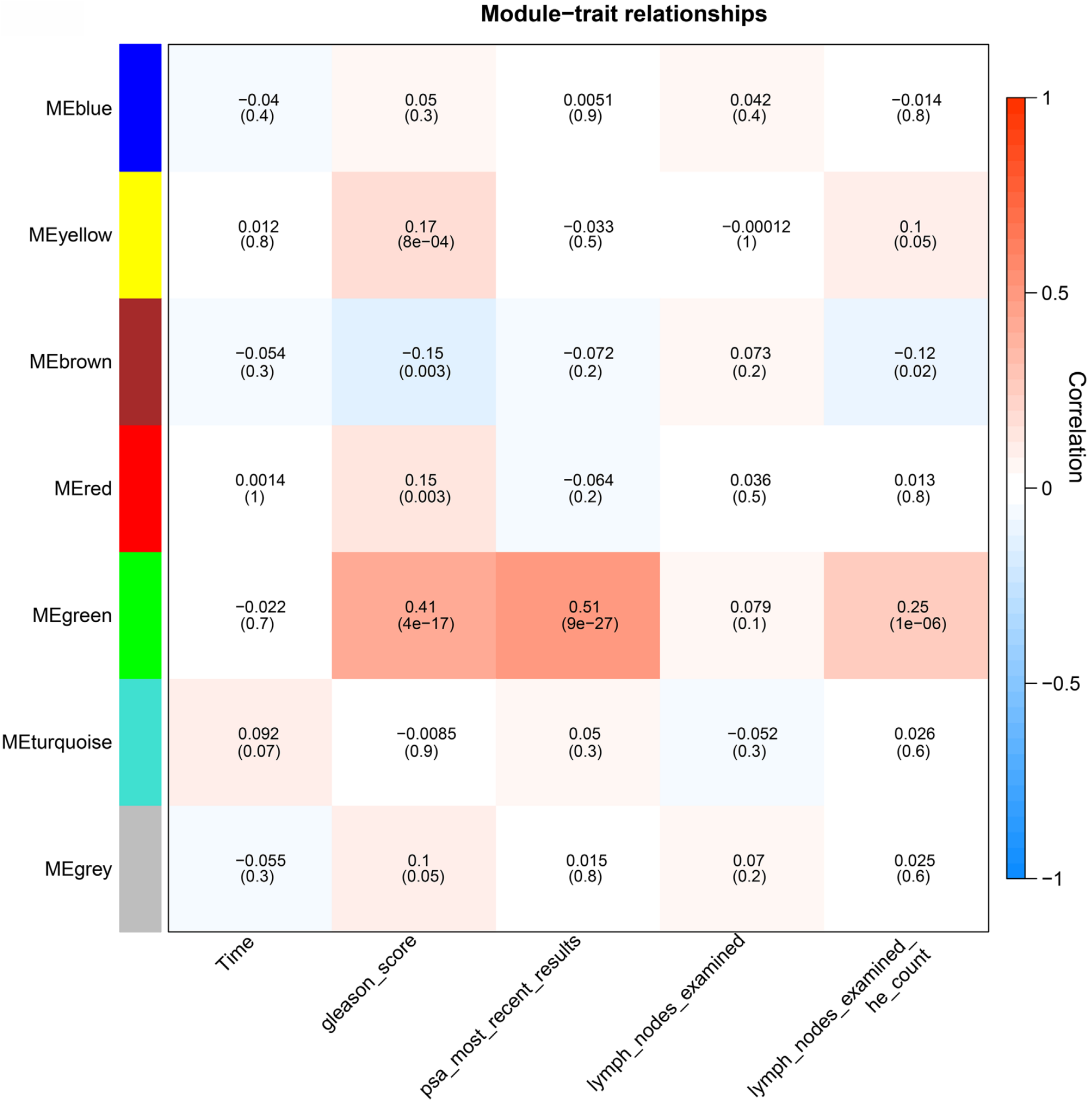
Module-trait associations Each row corresponds to a module eigengene, and each column corresponds to a pathoclinical parameter. The module eigengene is defined as the first principal component of a given module and considered a representative of the gene expression profiles in a module. Each grid contains the correlation value, calculated based on eigengene expression and clinical traits. The corresponding p-value is the Student asymptotic p-value for the correlation. The grid is color-coded by correlation according to the color bar of the correlation.

We further evaluated the relationship between these modules and the pathological parameters by calculating the correlation value of the eigengenes of each module (for a detailed definition, see the Materials and Methods section) with the clinical information obtained from the patients. The turquoise module was marginally significantly associated with survival time (p=0.07, Figure 2). The green module was associated with clinical parameters including Gleason score (p=4e-17), most PSA (p=9e-27) and lymph nodes according to haematoxylin and eosin (HE) staining (p=1e-06). Functional enrichment analysis based on KEGG pathways and biological process of Gene Ontology (GO) revealed the genes in the green module are involved in the GO term “cell cycle”. This observation is consistent with a previous report which also found cell cycle genes are correlated with PSA and the Gleason score [7]. The yellow module was also associated with Gleason score and lymph nodes according to HE examination. And as expected, the genes in this module were shown to participate in immune and defense responses. Finally, the brown module was negatively associated with Gleason score and lymph nodes according to HE examination. The genes assigned to this module significantly participate in focal adhesion pathways as well as the biological processes of muscle contraction and cell adhesion, consistent with previous studies on adhesion genes such as *CD44* [4], *CDH1* [5, 6] and *GJA1* [11].

### The turquoise module is correlated with survival time and associated with RNA-processing and protein translation

In the present study, we used survival time as one of the sample traits. There are two methods that are commonly used to identify prognostic markers. One method is at the gene module level, according to the correlation between the survival time and eigengene for each module. The other method is at the single gene level according to the correlation value (for details, see the Materials and Methods section) of gene expression and survival time.

**Figure 3:**
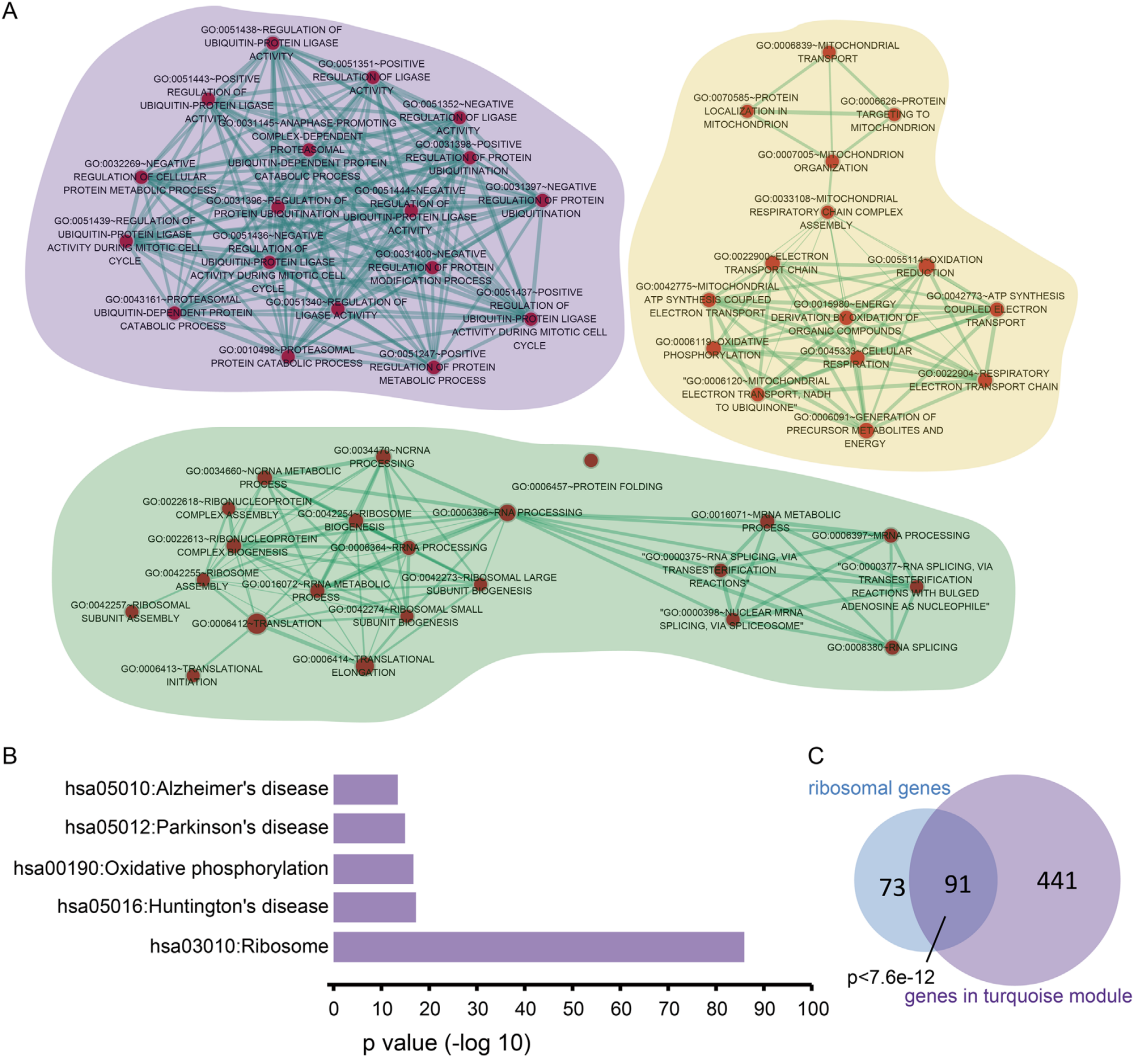
Genes in the turquoise module are involved in translation via ribosomal protein-coding genes **(A)** Enrichment analysis was performed for genes in the turquoise module. For the Gene Ontology BP terms, the Cytoscape app, Enrichment Map was used to identify the most correlated terms for genes in the turquoise module. One node represents one biological process. The node size increased with number of genes. The thickness of the edges between two terms is proportional to the similarity coefficient of the associated terms. **(B)** The enriched KEGG pathway of the genes in the turquoise module. **(C)** The overlapped genes between ribosomal protein-coding genes and those in the turquoise module. The p-value was calculated using the hypergeometric test.

With the most number of genes, the turquoise module was marginally significantly associated with survival time (p=0.07). Functional enrichment analysis revealed the genes in this module are associated with RNA processing and translation (Supplementary Table 1). As shown in Figure 3A, there are primarily 3 clusters of genes representing different biological functions in the turquoise module. The cluster with the purple background represents a translation-related function, while the clusters with the gold and sea green background are associated with mitochondrial-related processes and RNA processing, respectively. Further functional enrichment analysis revealed the genes in the turquoise module are significantly involved in the ribosome pathway and neurodegenerative diseases (Figure 3B).

In addition to the above functional enrichment analyses, we also performed a hypergeometric test on the turquoise module. The hypergeometric test is a widely-used method to identify the function of gene sets based on overlapping genes with known functions [12]. A gene family comprises a set of similar genes with similar biochemical functions. The HUGO gene nomenclature committee (HGNC) contains the members of each gene family. According to HGNC, the ribosomal protein family is comprised of 164 genes encoding for ribosomal proteins, including L ribosomal proteins (RPL), S ribosomal proteins (RPS) and mitochondrial ribosomal proteins (MRPL, MRPS) [13]. As shown in Figure 3C, the genes in the turquoise module significantly overlapped with the HGNC ribosomal protein family (hypergeometric test, p<7.6e-12). Taken together, our results show the turquoise module is correlated with survival time and closely associated to RNA processing and protein translation.

### *FDZ7* and *MEIS1* are good prognostic markers for PCa patient survival time in gleason score-related modules

Gene significance (GS) is a measure to quantify the correlation of individual genes with clinical information [14]. Similarly, for individual genes, module membership (MM) is a measure to evaluate the degree of correlation between the module eigengene and the expression level of a single gene [15]. In this study, survival time was used as the clinical information. At the single gene level, prognostic markers can be identified using the correlation of gene expression and survival time. Genes with high GS and MM are regarded as the most important components of the modules, which are remarkably correlated with survival time. Among the genes in modules which are notably linked to Gleason score, we identified genes associated with high GS and high MM.

Our current findings show there are four modules which are correlated with Gleason score: the yellow, red, green and brown modules (Figure 2). Considering the high soft threshold β=22, we used the cut-off GS>0.1 [15, 16] and MM>0.8 [17] to determine which genes are critical for survival time in these four modules. Only 9 genes (*FZD7*, *PRTFDC1*, *FAXDC2*, *MEIS1*, *ST5*, *FBXL22*, *EOGT,* and *NPR2*) in the brown module met the criteria. In the brown module, the co-expression network comprised of 296 nodes and 5698 edges with adjacency>0.02. Among the 9 genes identified, *FZD7*, *FBXL22,* and *MEIS1* were the top 3 ranked genes based on the number of interacting genes.

**Figure 4:**
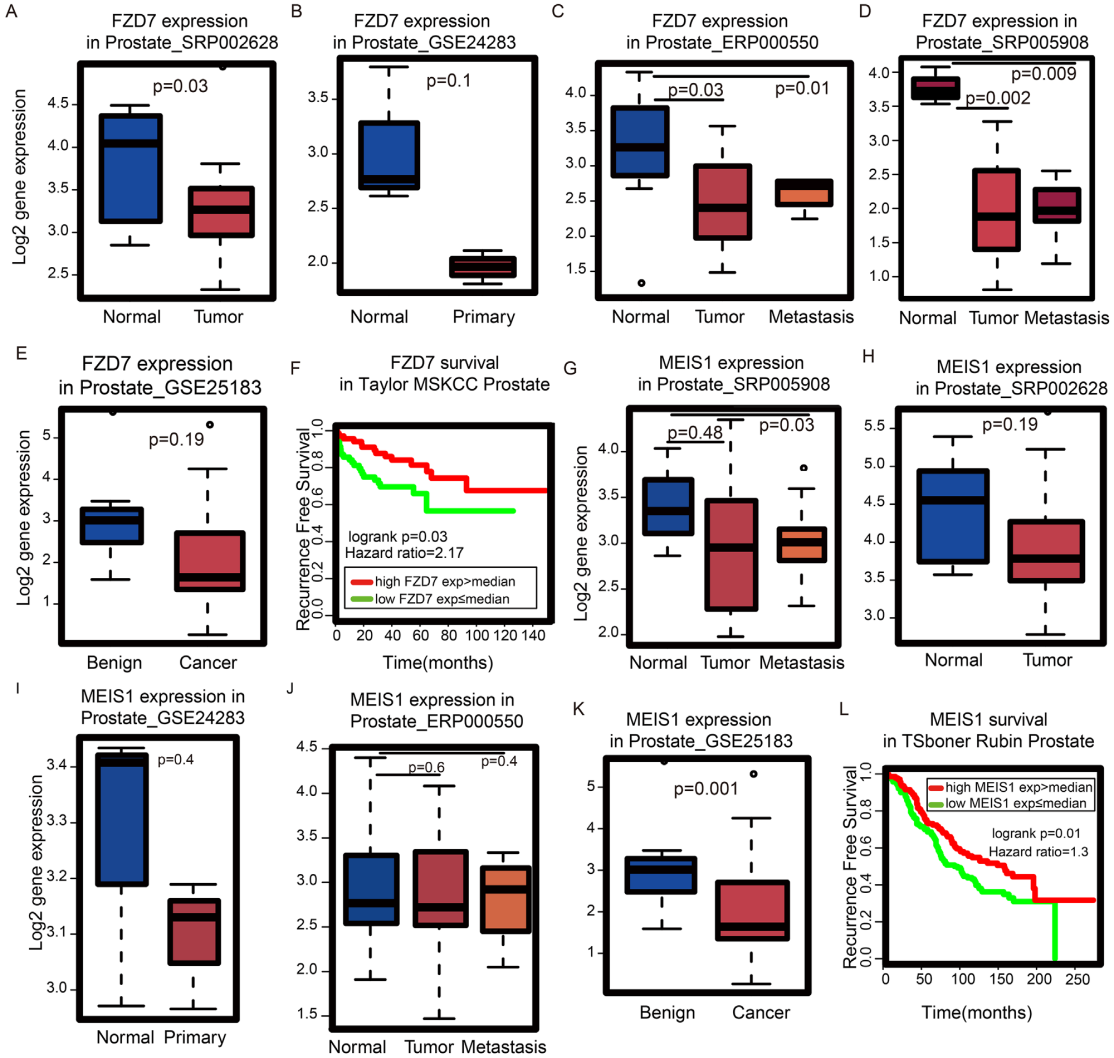
Identification of genes important for survival time in Gleason score-related modules **(A)** *FZD7* is down-regulated in tumor vs. normal in dataset SRP002628 from publication [PMID: 21571633], **(B)** GSE24283 from publication [PMID: 21261984], **(C)** ERP000550 from publication [PMID: 22349460], **(D)** SRP005908 from publication [PMID: 21036922] and **(E)** GSE25183 from publication [PMID: 21804560]. **(F)** According to the Kaplan-Meier plot, patients with high *FZD7* expression have better survival probability using Taylor’s MSKCC dataset. **(G)** *MEIS1* is down-regulated in tumor vs. normal in dataset SRP005908 from publication [PMID: 21036922], **(H)** SRP002628 from publication [PMID: 21571633], **(I)** GSE24283 from publication [PMID: 21261984], **(J)** ERP000550 from publication [PMID: 22349460] and **(K)** GSE25183 from publication [PMID: 21804560]. **(L)** According to the Kaplan-Meier plot, patients with high expression of *MEIS1* expression have better survival probability using Tsboner Rubin’s dataset.

Next, we examined the expression pattern of *FZD7*, *FBXL22,* and *MEIS1* in several PCa cohorts to establish whether any of these genes could be potential biomarkers. *FBXL22* is lowly expressed in the TCGA prostate cancer dataset (average FPKM=1.8, SD=1.2) and therefore may not be a good biomarker candidate. *FZD7* is a member of the Frizzled receptor family and has been shown to be important in cancer development and progression by activating Wnt pathways [18]. Although *FZD7* is up-regulated in multiple tumors, including colorectal cancer and breast cancer [18], we found *FZD7* i*s* down-regulated in PCa relative to normal tissue across multiple cohorts (Figure 4A-4D). Similar observations were also found in PCa cell lines (Figure 4E). Moreover, patients with high *FZD7* expression have better disease-free survival rates (Figure 4F). *MEIS1* is a novel *AR* co-repressor [19]. Similar to *FZD7*, we found *MEIS1* is down-regulated in both prostate tumors (Figure 4G-4J) and PCa cell lines (Figure 4K), and a high *MEIS1* expression is an indication of better overall survival for PCa patients (Figure 4L) [20]. Taken together, our GS and MM analysis have revealed *FZD7* and *MEIS1* as potentially new prognostic genes for PCa that are associated with good patient outcome.

### Identification of novel prognostic lncRNAs in PCa

**Figure 5:**
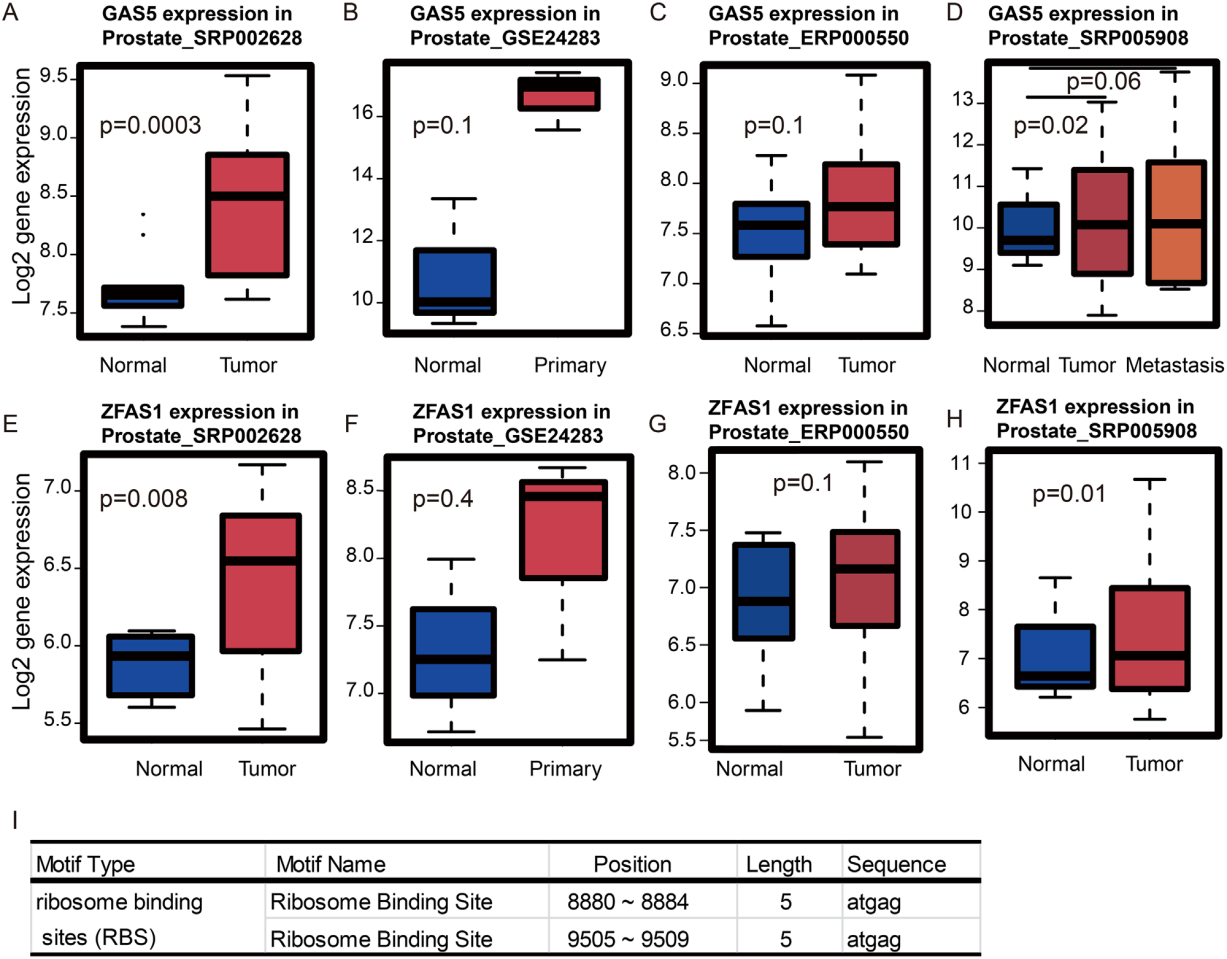
Cross-dataset expression of survival related lncRNAs **(A)** *GAS5* is up-regulated in tumors vs. normal tissues in dataset SRP002628 from publication [PMID: 21571633], **(B)** GSE24283 from publication [PMID: 21261984], **(C)** ERP000550 from publication [PMID: 22349460] and **(D)** SRP005908 from publication [PMID: 21036922]. **(E)** *ZFAS1* is up-regulated in tumor vs. normal tissues in dataset SRP002628 from publication [PMID: 21571633], **(F)** GSE24283 from publication [PMID: 21261984], **(G)** ERP000550 from publication [PMID: 22349460]and **(H)** SRP005908 from publication [PMID: 21036922]. **(I)** RegRNA identified the ribosome-binding sites of *ZFAS1*.

The module that was most significantly associated with survival time was the turquoise module. Recently, a number of lncRNAs have been implicated in PCa biology. For example, *PCAT-1*, *PRNCR1,* and *MALAT1* were shown to regulate the development and progression of PCa [21–23]. Therefore, we decided to see whether there are any potential prognostic lncRNAs in the turquoise module. Notably, we found four lncRNAs including *NCBP2-AS2, LINC00116*, *GAS5,* and *ZFAS1*. *NCBP2-AS2* did not show any expression differences between normal and tumor tissues in PCa (data not shown), however, it has been reported to be up-regulated in lung squamous cell carcinoma compared to lung adenocarcinoma [24]. *LINC00116* is up-regulated in PCa relative to normal tissue (data not shown), however, the function of *LINC00116* has not been explored yet. For *GAS5 and ZFAS1*, both lncRNAs are also up-regulated in PCa relative to normal prostate tissues in the four datasets that we examined (Figure 5).

As shown above, the genes in the turquoise module are highly associated with biological functions related to ribosomes. Therefore, we asked whether *LINC00116*, *ZFAS1* or *GAS5* could be directly involved in the translation process. To address this, we used the web tool, RegRNA, which looks for ribosome binding sites (RBS) in RNA sequences [25]. As shown in Figure 5I, *ZFAS1* but not *LINC00116* or *GAS5* contains RBS. This finding suggests that *ZFAS1* may be directly involved in ribosome-related translation.

**Figure 6:**
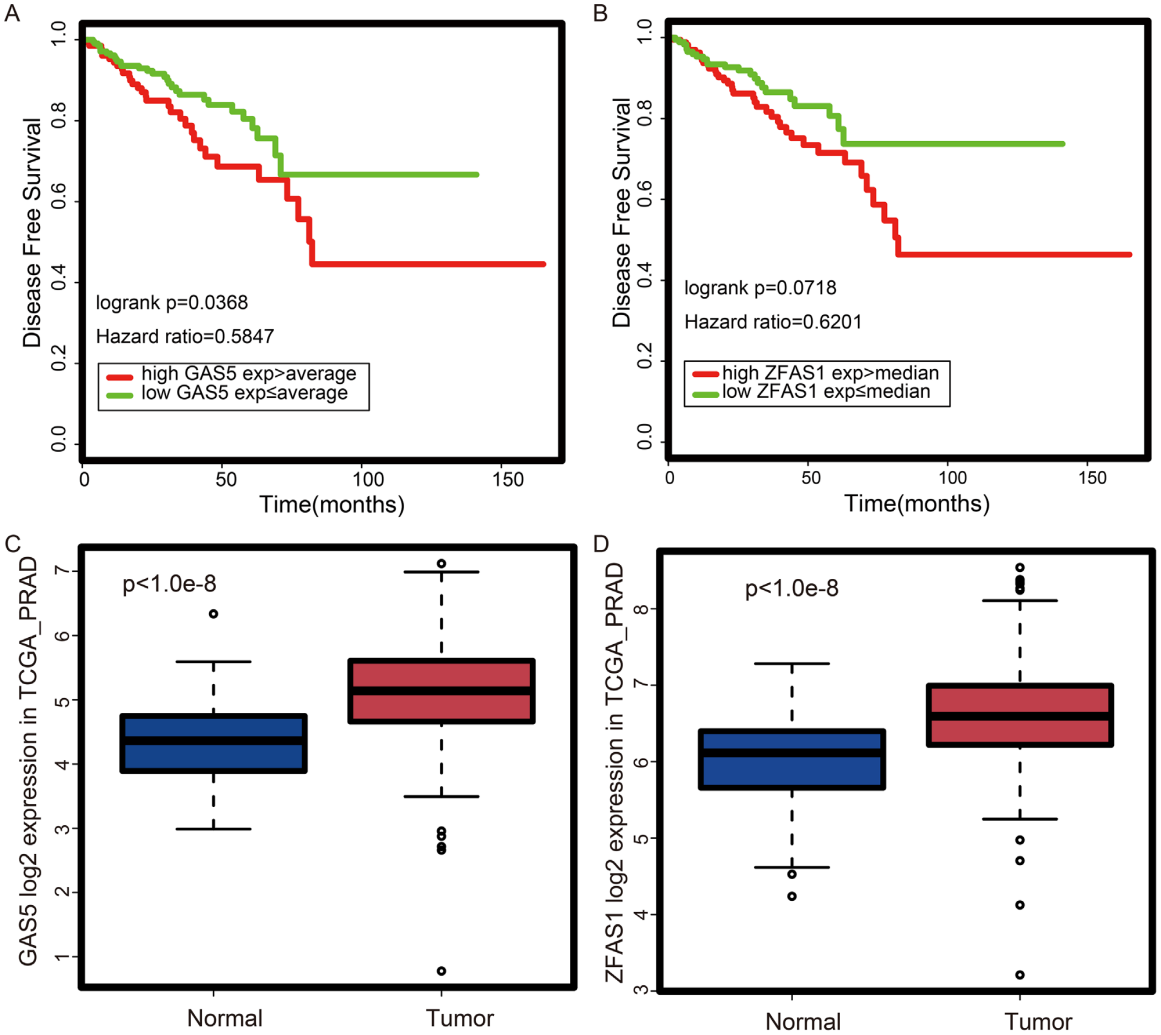
Expression and prognostic potential of *GAS5* and *ZFAS1* according to TCGA prostate cancer dataset **(A)** According to the Kaplan-Meier plot, patients with high *GAS5* expression have worse survival probability. **(B)** According to the Kaplan-Meier plot, patients with high *ZFAS1* expression have worse survival probability. **(C)** *GAS5* is up-regulated in tumor vs. normal tissues. **(D)** *ZFAS1* is up-regulated in tumor vs. normal tissues.

Next, we assessed the prognostic potential of *LINC00116*, *GAS5,* and *ZFAS1*. For this, we performed survival analysis with a log-rank test to determine whether patients with high and low expression levels of these lncRNAs have significantly different survival rates. As shown in Figure 6A-6B, the high expression of *GAS5* or *ZFAS1* is correlated with a worse outcome in PCa. These results (Figure 6A-6B) are consistent with the data obtained from TANRIC (Supplementary Figure 1) [26]. In contrast, *LINC00116* appears not to be a good predictor of patient outcome. Thus, we further focused on *GAS5* and *ZFAS1*, which are up-regulated in PCa tissues relative to normal samples (Figure 5 and Figure 6C-6D). Since the turquoise module is associated with RNA-processing and protein translation (Figure 3), we examined the expression correlation between ribosomal genes, *GAS5* and *ZFAS1*. In Pearson’s correlation coefficient (PCC) analysis, both *ZFAS1* (0.5<PCC<0.82, p<1.0e-9) (Supplementary Figure 2) and *GAS5* (0.41<PCC<0.84, p<1.0e-9) (Supplementary Figure 3) are correlated significantly with ribosomal genes. Based on the above results, we believe *GAS5* and *ZFAS1* are potent novel prognostic lncRNAs in PCa that have a role in protein translation.

### The interaction network of *miR-940* and lncRNAs in PCa

**Figure 7:**
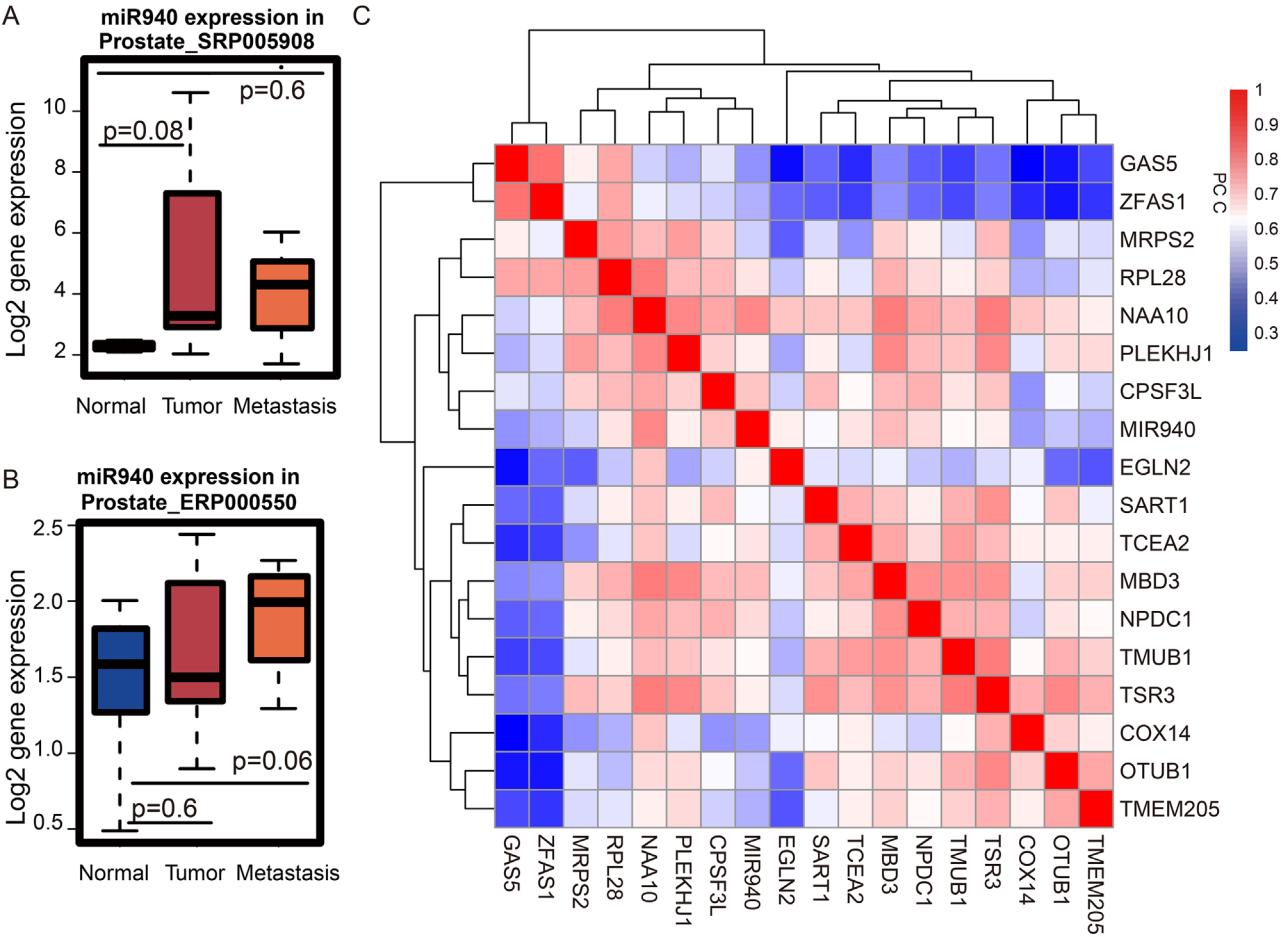
Expression of miRNA and its targets in the turquoise module **(A)** *MiR-940* is up-regulated in tumor vs. the normal in dataset SRP005908 from publication [PMID: 21261984], **(B)** ERP000550 from publication [PMID: 22349460]. **(C)** Based on the gene expression profile from TCGA prostate cancer, the expression similarity of the genes of interest is shown in the two-way clustering heat map. The interested genes include two lncRNAs (*GAS5* and *ZFAS1*), one miRNA (*miR-940*) and target genes of *miR-940* in the turquoise module. *GAS5* and *ZFAS1* are highly correlated with each other.

The reciprocity among miRNAs, lncRNAs, and protein-coding genes constitute an intricate interaction network, which is dysregulated in all types of human cancers [27]. To dissect this complex network, we began by exploring the role of miRNAs in the turquoise module. We found microRNA *miR-940* in the turquoise module. *MiR-940* is up-regulated in both primary and metastatic PCa patients (Figures 7A-7B). According to DIANA-miRPath [28], the targets of *miR-940* are significantly enriched in “prostate cancer” (p=0.045). Moreover*, miR-940* has been shown to suppress PCa migration and invasion by regulating the expression of *MIEN1* [29]. To determine whether *miR-940* could potentially regulate the expression of lncRNAs in the turquoise module, we used LncBase [30] which hosts a database of non-coding RNA targets of microRNA. Surprisingly, *miR-940* has been experimentally validated by immunoprecipitation assays to interact with both *GAS5* and *ZFAS1* [31, 32]. Taken together, we speculate that the *GAS5/ZFAS1/miR-940* axis plays key roles in PCa via the protein translation pathway. How these three factors influence the outcome of PCa patients remains elusive.

MiRNAs bind to partially complementary sequences of their target mRNAs, and many of these molecules have been widely implicated in various human diseases. Thus, to understand the relationship between *miR-940* and its target genes in the turquoise module and how they are integrated as part of the *GAS5/ZFAS1/miR-940* axis, we searched for mRNA targets of *miR-940*. For this, we used TarBase [33], a data warehouse that stores targets of miRNAs originating from both manual curation and experimental studies. From our search, we identified 15 gene targets of *miR-940* belonging to the turquoise module, including *COX14*, *CPSF3L*, *EGLN2*, *MBD3*, *MRPS2*, *NAA10*, *NPDC1*, *OTUB1*, *PLEKHJ1*, *RPL28*, *SART1*, *TCEA2*, *TMEM205*, *TMUB1*, and *TSR3*. Next, to obtain additional information on how these genes are connected with *miR-940* as well as *GAS5* and *ZFAS1*, we obtained the expression information for these 15 genes along with *miR-940*, *GAS5,* and *ZFAS1* and performed PCC analysis. Based on the correlation heat map for these genes, *ZFAS1* and *GAS5* are highly positively correlated with each other (PCC=0.83, p<1.0e-9, Figure 7C). Moreover, correlation analysis also revealed *miR-940* is positively correlated with *GAS5* (PCC=0.47) and *ZFAS1* (PCC=0.49) (Figure 7C) which suggests *miR-940* also likely to be involved in prostate cancer.

**Figure 8:**
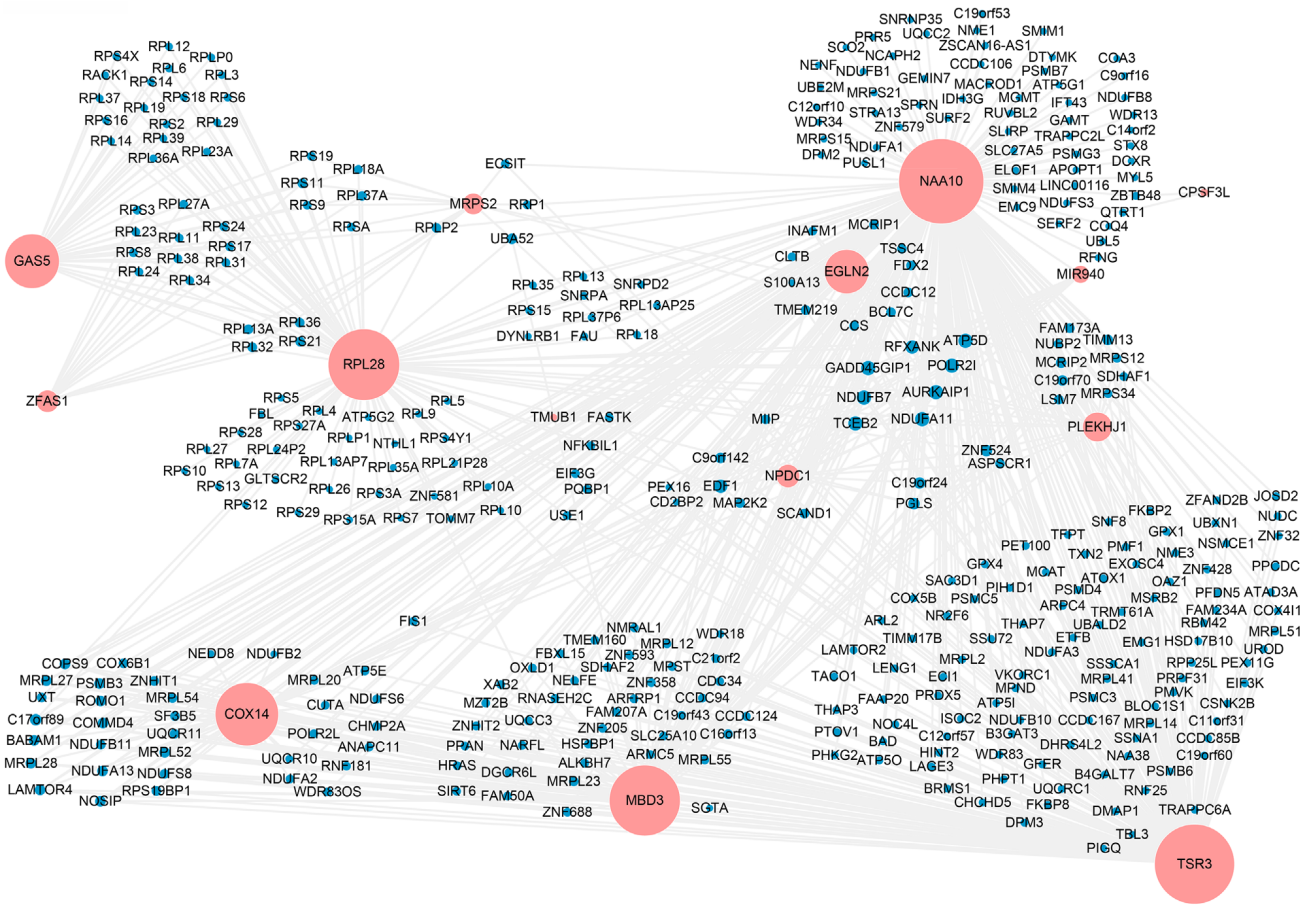
Co-expression network of *GAS5*, *ZFAS1*, *miR-940* and target genes of *miR-940* in the turquoise module The co-expression network was constructed for the genes of interest which included two lncRNAs (*GAS5* and *ZFAS1*), one miRNA (*miR-940*), target genes of *miR-940* in the turquoise module and their co-expressed genes with correlation coefficients larger than 0.01. Node size is proportional to the number of co-expressed genes.

Finally, to further dissect the role of *GAS5* and *ZFAS1* in PCa, we created a gene co-expression subnetwork for the genes in the turquoise module, which included *GAS5*, *ZFAS1*, *miR-940* and its targets from TarBase (Figure 8). As shown in the network, *GAS5* and *ZFAS1* share many co-expressed genes, including genes encoding both S and L ribosomal proteins. One of the target genes of *miR-940* is *NAA10* which interacts with the most genes in the network. Interestingly, both *GAS5* and *ZFAS1* are linked to *MRPS2*, a gene coding for mitochondrial ribosomal proteins which is also a target of *miR-940* via *NAA10*. *RPL28*, as the third largest node in the network and a target of *miR-940*, interacts with *GAS5* and *ZFAS1* via other ribosomal protein-encoding genes. A simplified version of the gene co-expression network using the Cytoscape app, ThematicMap, can be found in Supplementary Figure 4. Again, this network map shows *GAS5* and *ZFAS1* are targeted by *miR-940* via *NAA10* and *RPL28* and possibly other targets of *miR-940* as well (genes in nodes 3 and 4, including ribosomal genes). In summary, our results show *miR-940* indirectly targets *GAS5* and *ZFAS1* via its mRNA targets, including *NAA10* and *RPL28*.

## DISCUSSION

In this study, we performed co-expression gene network analysis on PCa patient RNA-seq samples and identified a gene module that correlated with patient survival time. In functional enrichment analysis, we showed the genes in this module are involved in translation and RNA-processing. Translation pathways have previously been implicated to predict patient survival in PCa. For example, higher expression of *EIF4E*, a family member of the eukaryotic translation initiation factor, is associated with worse outcome in PCa patients [34, 35]. Moreover, it is now known that RNA processing contributes to the generation of androgen receptor splice variants, the constitutive activation of which is associated with poor prognosis [36].

Due to its tissue-specific and cancer-specific expression, long non-coding RNAs are favorable candidates as diagnostic or prognostic biomarkers for cancer. Indeed, a number of lncRNAs have emerged as potential biomarkers for PCa. High *SCHLAP1* expression in PCa has been reported to predict worse patient outcome [37–40]. The up-regulation of *UCA1* [41] and *NEAT1* [42] also indicates a poor prognosis in patients suffering from PCa. In contrast, a low *PCAT29* expression has been shown as an indicator of a higher potential for recurrence [43]. Similarly, the down-regulation of *PCAT14* [44, 45] and *DRAIC* [46] are both associated with poor prognosis of PCa. In this work, we identified several non-coding RNAs in the module that correlated with survival time, including *GAS5*, *miR-940,* and *ZFAS1*. Furthermore, our findings suggest that *GAS5* and *ZFAS1* are potential novel prognostic markers for PCa.

*GAS5* expression and its clinical implication have been examined in many types of cancers. For example, *GAS5* is down-regulated in breast cancer [47]. In squamous cell carcinoma of the head and neck, higher expression of *GAS5* in patients indicates higher recurrence-free survival [48]. Functionally, *GAS5* has been shown to bind to the DNA-binding domain of *AR* [49]. This is because part of the *GAS5* sequence is similar to the glucocorticoid receptor responsive element [50]. Therefore, *GAS5* can prevent the binding of AR to its target DNA sequences by sequestering the androgen/*AR* complex [49]. In PCa, *GAS5* has been shown to promote apoptosis [51] and inhibit cell proliferation and cancer progression by targeting *miR-103* and the mTOR pathway [52]. In the present study, our results suggest that *GAS5* may also be involved in regulating protein translation in PCa and a high *GAS5* expression is a predictor of worse disease-free survival.

The expression of *ZFAS1*, like *GAS5,* is also up-regulated in normal mammary glands compared to breast cancer tissues [53]. Our current results show that high *ZFAS1* expression is an indicator of lower disease-free survival for PCa patients. This predictive power of *ZFAS1* does not appear to be limited to PCa, as it has been reported for gliomas as well [54]. With regards to function, *ZFAS1* has been shown to regulate cell proliferation and migration of ovarian cancer by targeting *miR-150-5p* [55], whereas, in gastric cancer, *ZFAS1* was demonstrated to accelerate cell proliferation via repressing the expression of *KLF2* and *NKD2* [56]. In this study, we also showed *ZFAS1* may have functions related to protein translation which has been previously reported for breast cancer [53].

Compared to *GAS5* and *ZFAS1*, the role of *miR-940* is less clear and appears to be different depending on the type of cancer. Moreover, in some cancer types, the finding has even been contradictory. In general, *miR-940* has been reported as a tumor suppressor in many studies. In addition, it is highly expressed in normal tissues compared with tumors in nasopharyngeal carcinoma [4], breast cancer [4], pancreatic ductal adenocarcinoma [57], ovarian cancer [58], hepatocellular carcinoma and gastric cancer [59]. On the other hand, *miR-940* has also been reported as an oncogene with higher expression in tumor compared to normal tissues in pancreatic cancer [60], oral tongue squamous cell carcinoma, cervical cancer [4] and gastric cancer [60]. Currently, studies of *miR-940* in cancer are still sparse and contradictory. For example, in one study *miR-940* was reported to act as an oncogene in gastric cancer by directly down-regulating *ZNF24* expression [60]. But in another study also on gastric cancer, *miR-940* was reported as a tumor suppressor [59]. Therefore, the difference in expression trend of *miR-940* may lie in the different cohorts and cancer types. Further studies are needed to clarify these observations.

Here, we described the regulation between *GAS5*, *ZFAS1,* and *miR-940* in PCa. Our findings suggest *miR-940* directly targets *NAA10* and indirectly targets *ZFAS1* and *GAS5* via *MRPS2* and other ribosomal genes. In addition, we used WGCNA to detect gene modules that are significantly associated with pathoclinical parameters. We also identified prognostic biomarkers based on correlations between survival time and gene expression from both the single gene and gene module perspectives. Finally, we inferred the function of non-coding RNAs based on co-expressed genes. In conclusion, our work suggests that co-expression analysis of large-scale RNA-seq profiling can facilitate the identification and functional characterization of novel prognostic markers.

## MATERIALS AND METHODS

### Data acquisition

HTSeq-FPKM TCGA expression profiling was downloaded from https://gdc-portal.nci.nih.gov/projects/t for PCa, together with clinical data, including “BCR status”, “tumor status”, “Gleason score”, “pathologic N”, “pathologic T”, “psa_most_recent_results”, “lymph_nodes_examined”, “lymph_nodes_examined_he_count” and “residual tumor”. Clinical information was obtained from 380 patients, and the following analyses were performed on these patients.

### Gene co-expression network construction and module identification

WGCNA was used to create gene co-expression networks and to identify gene modules [14, 61]. All transcripts expressed (FPKM>1) in at least half of the patients were included for WGCNA. First, a symmetric matrix of Pearson’s correlation was computed between all gene pairs. Second, the correlation matrix was raised to power β = 30 to obtain the adjacency matrix. Considering its characteristic of scale-free topology (R^2^ = 0.9), the power β=22 is selected to construct the adjacency network (Figure 1). The adjacency matrix was further transformed to a topological overlap matrix (TOM), which aims to evaluate the most strongly correlated genes. The matrix (1-TOM) was used for hierarchical clustering. In the hierarchical dendrogram, its branches are regarded as the gene modules, which are cut using branch cutting algorithms [62]

The gene significance (GS) of the *i*th gene can be defined: $GS_{i}={\left|cor\left(x_{i},T\right)\right|}^{\beta }$, where $x_{i}$ is the expression profile of gene *i* and *T* is the sample trait. β=22 (Figure 1A) is the power we used to find gene modules. For each module, the module eigengene was represented by the first principal component of the expression profile. Modules were merged together when the module eigengenes are highly correlated (correlation > 0.75). The module-trait relationships (Figure 2) exhibits the correlation of eigengene expression in a module $q(E^{\left(q\right)})$ and clinical traits T (survival time, Gleason score, PSA and number of lymph nodes). The correlation value in each grid was calculated as ${\left|cor\left(E^{\left(q\right)},T\right)\right|}^{\beta }$, with corresponding Student asymptotic p-value. The module membership (MM) quantifies the extent of similarity of a pair of gene and module. MM of each gene was calculated as ${MM}^{q}\left(i\right)=cor\left(x_{i},E^{q}\right)$. For more details, please refer to [63]. The correlation network of genes in the turquoise module was constructed based on adjacency threshold as 0.01.

### Functional enrichment analysis

Fisher’s exact test was adopted to measure the gene enrichment in the annotation terms according to DAVID [64, 65]. When the Bonferroni-adjusted p≤ 0.05 was used, we assumed that the user gene lists were significantly enriched in this functional term. “Enrichment Map”, a Cytoscape plugin (http://cytoscape.org/) [66], was used to identify the function clusters for genes in the largest module (turquoise module), facilitating the interpretation of the enrichment terms.

## SUPPLEMENTARY MATERIALS FIGURES AND TABLE



## Abbreviations

(lncRNAs): Long non-coding RNAs
(ncRNAs): non-coding RNAs
(miRNAs): microRNAs
(WGCNA): weighted correlation network analysis
(PCa): Prostate cancer
(PSA): prostate-specific antigen
(CCP): cell cycle progression
(HE): hematoxylin and eosin
(HGNC): HUGO gene nomenclature committee
(RPL): L ribosomal proteins
(RPS): S ribosomal proteins
(GS): Gene significance
(MM): module membership
(TOM): topological overlap matrix
(RBS): ribosome binding sites
(PCC): Pearson’s correlation coefficient
