# Supplementary material for: Long non-coding RNA GAS5 and ZFAS1 are prognostic markers involved in translation targeted by miR-940 in prostate cancer
Source: Oncotarget. 2017 Dec 14;9(1):1048–62. doi: 10.18632/oncotarget.23254 (PMC5787418; doi:10.18632/oncotarget.23254)
Supplement: Supplementary file 1 [file oncotarget-09-1048-s001.pdf]

Long non-coding RNA *GAS5* and *ZFAS1* are prognostic markers involved in translation targeted by *miR-940* in prostate cancer

SUPPLEMENTARY MATERIALS

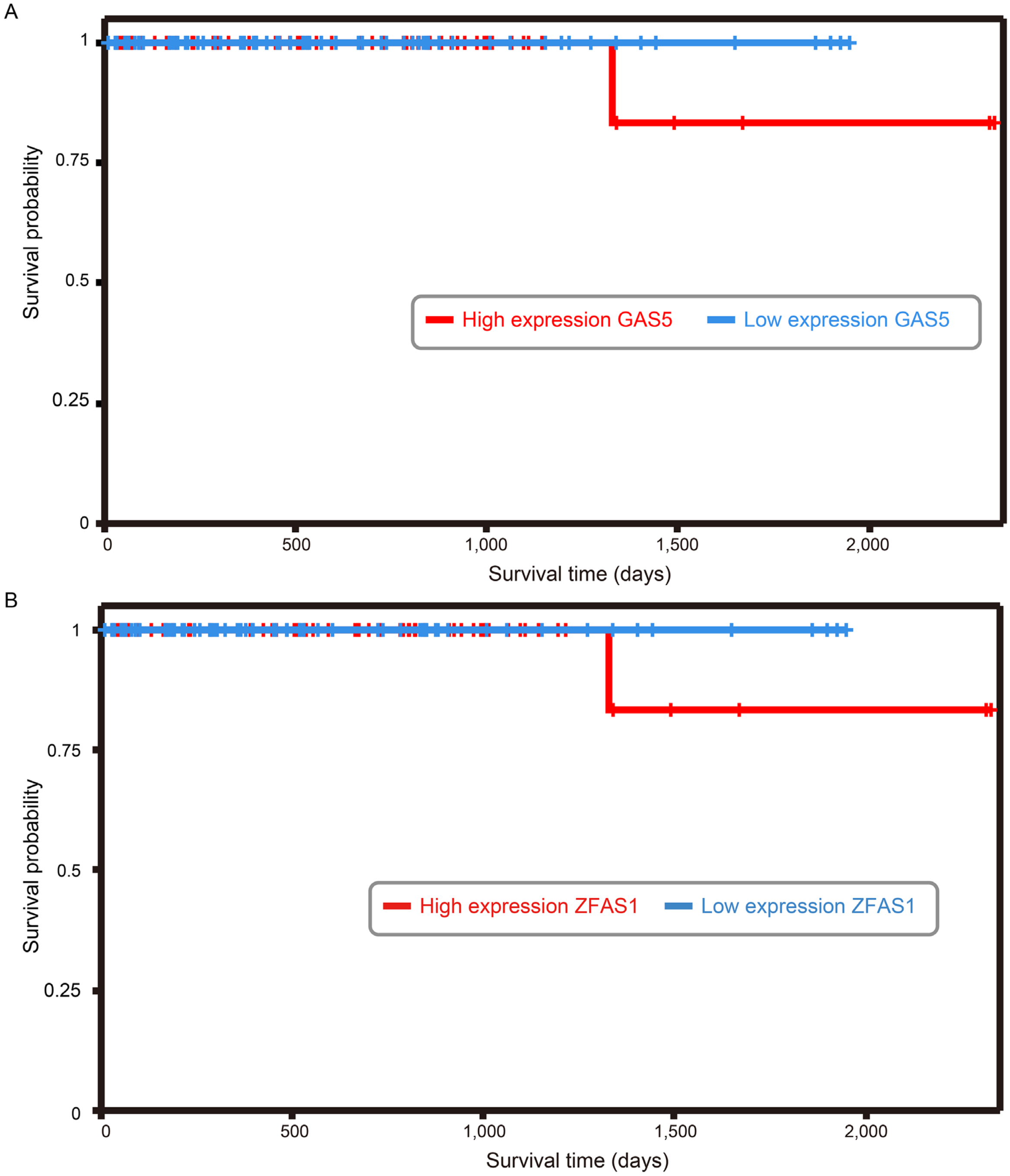

Supplementary Figure 1: Overall survival of *ZFAS1* and *GAS5* based on TANRIC. **(A)** Patients with high *GAS5* expression have worse survival probability. **(B)** Patients with high *ZFAS1* expression have worse survival probability.

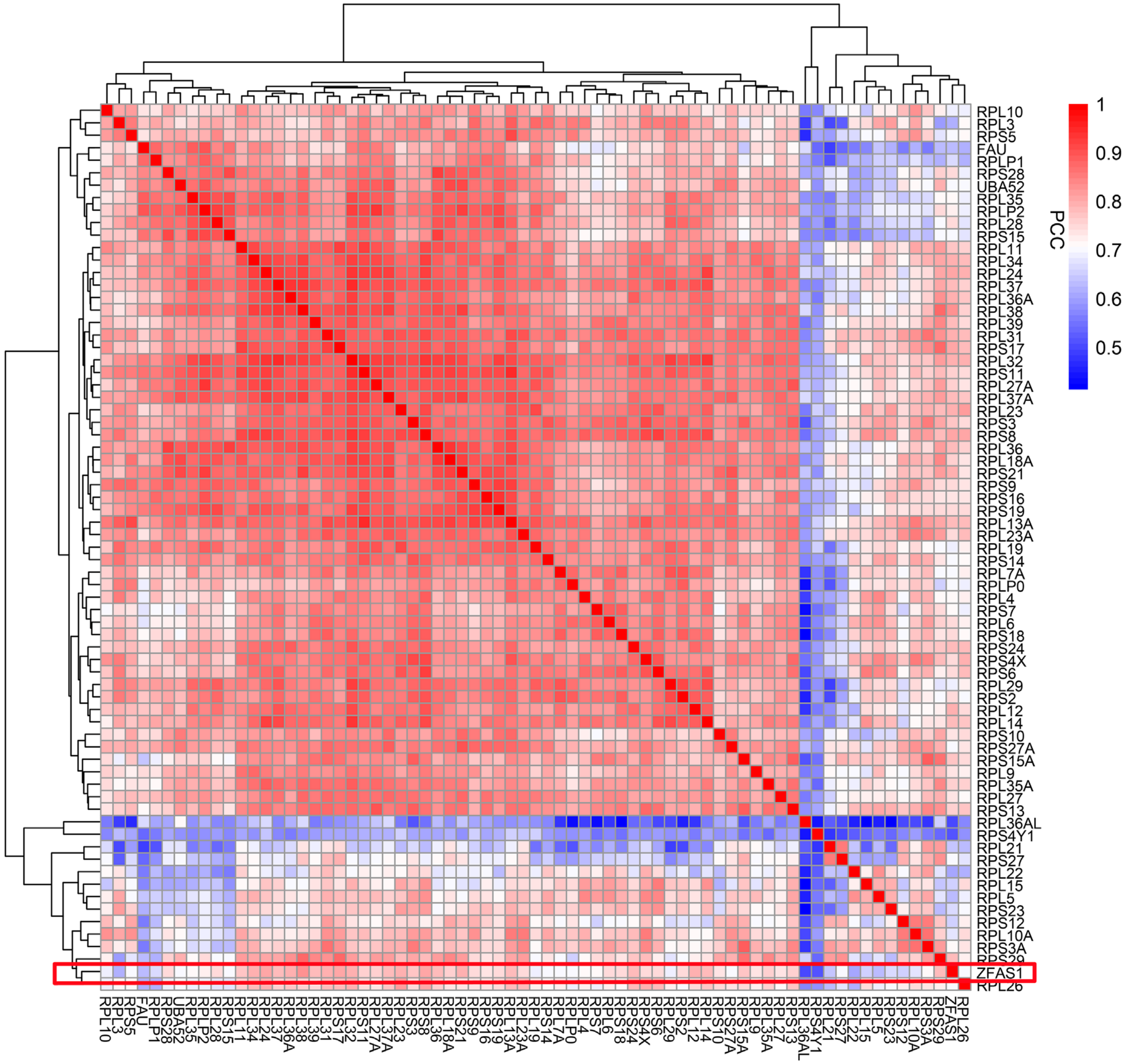

Supplementary Figure 2: Expression correlation between *ZFAS1* and ribosome genes in the turquoise module. The correlation is calculated using Pearson's correlation coefficient (PCC). *ZFAS1* is significantly correlated with ribosomal genes. (0.5<PCC<0.82, p<1e-9).

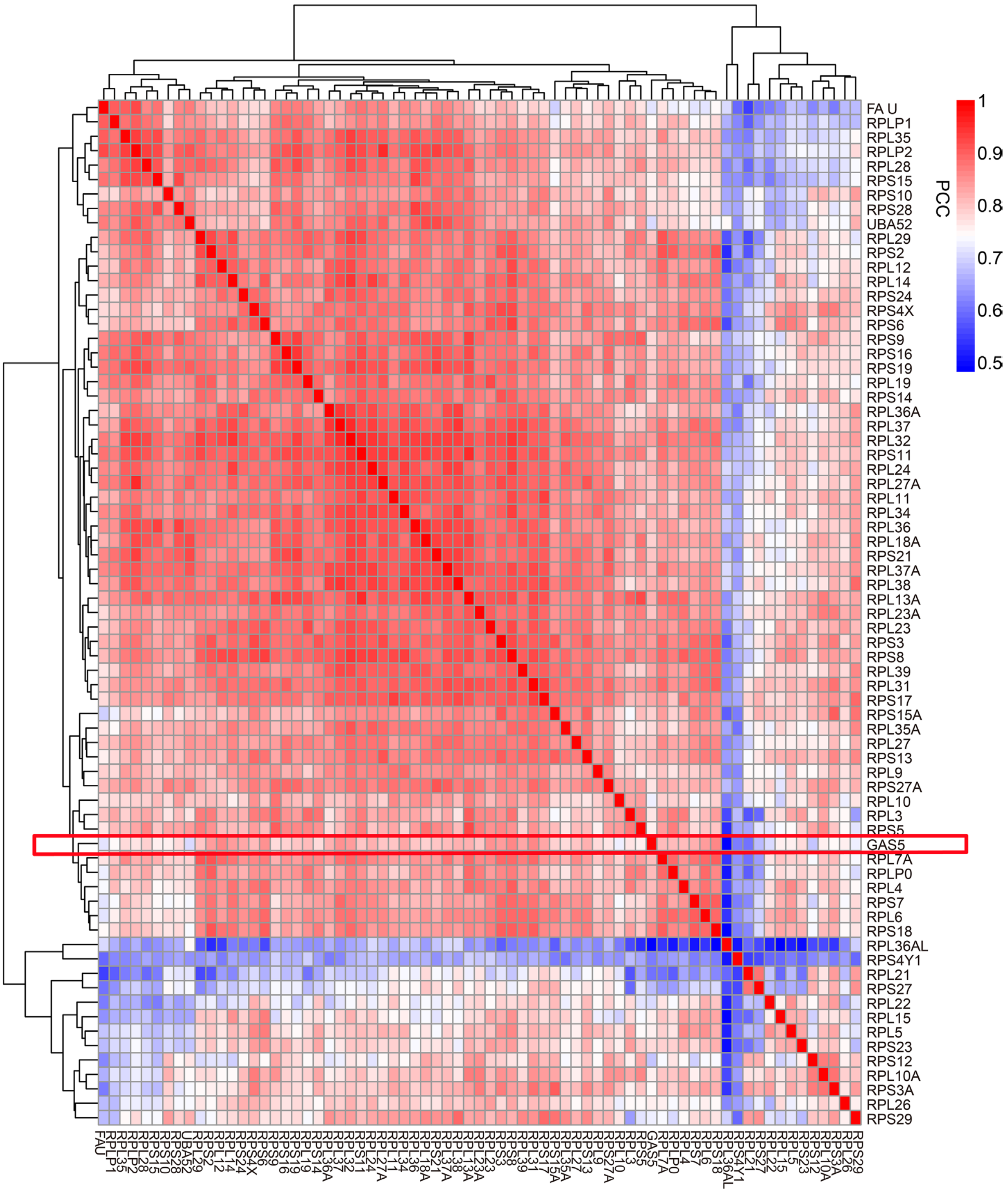

Supplementary Figure 3: Expression correlation between *GAS5* and ribosome genes in the turquoise module. The correlation is calculated using Pearson's coefficient (PCC). *GAS5* is significantly correlated with ribosomal genes. (0.5<PCC<0.82, p<1e-9).

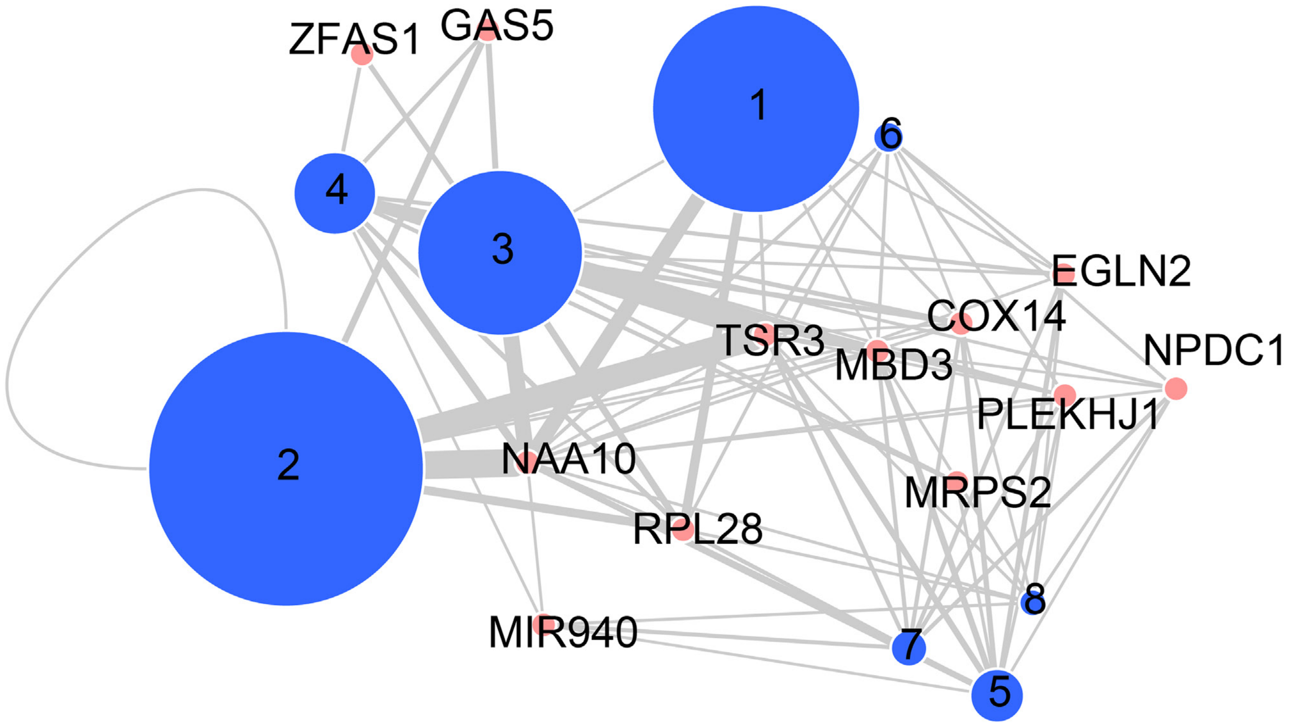

Supplementary Figure 4: Simplified co-expression network of *GAS5*, *ZFAS1*, *miR-940* and target genes of *miR-940* in the turquoise module. The co-expression network of the genes of interest was simplified using ThematicMap, which is a Cytoscape app used to simplify and visualize the complex gene co-expression network. Node 1 to 8 represents a gene set, respectively.

Supplementary Table 1: Biological processes enriched by genes in the turquoise module according to enrichment analysis on Gene Ontology

Term	Count	PValue	Adjusted pvalue
GO:0006412~translation	96	1.36E-72	1.86E-69
GO:0006414~translational elongation	72	8.76E-90	1.20E-86
GO:0006396~RNA processing	54	3.91E-16	6.08E-13
GO:0055114~oxidation reduction	46	3.56E-09	4.87E-06
GO:0006091~generation of precursor metabolites and energy	40	1.14E-15	1.52E-12
GO:0022613~ribonucleoprotein complex biogenesis	30	7.41E-15	1.02E-11
GO:0006119~oxidative phosphorylation	29	1.97E-21	2.69E-18
GO:0022900~electron transport chain	29	1.75E-19	2.39E-16
GO:0034470~ncRNA processing	29	1.45E-13	1.99E-10
GO:0034660~ncRNA metabolic process	29	2.51E-11	3.43E-08
GO:0042254~ribosome biogenesis	26	1.66E-15	2.28E-12
GO:0015980~energy derivation by oxidation of organic compounds	26	9.69E-14	1.33E-10
GO:0045333~cellular respiration	25	5.93E-17	1.52E-13
GO:0042775~mitochondrial ATP synthesis coupled electron transport	23	1.54E-20	2.10E-17
GO:0042773~ATP synthesis coupled electron transport	23	1.54E-20	2.10E-17
GO:0022904~respiratory electron transport chain	23	4.69E-19	6.42E-16
GO:0008380~RNA splicing	23	9.62E-06	1.31E-02
GO:0006397~mRNA processing	23	6.29E-05	8.26E-02
GO:0006364~rRNA processing	22	3.36E-14	4.59E-11
GO:0016072~rRNA metabolic process	22	8.30E-14	1.14E-10
GO:0006120~mitochondrial electron transport, NADH to ubiquinone	19	7.54E-18	1.03E-14
GO:0031396~regulation of protein ubiquitination	13	1.58E-05	2.15E-02
GO:0051443~positive regulation of ubiquitin-protein ligase activity	12	2.53E-06	3.46E-03
GO:0051351~positive regulation of ligase activity	12	3.87E-06	5.29E-03
GO:0031397~negative regulation of protein ubiquitination	12	4.44E-06	6.06E-03
GO:0051438~regulation of ubiquitin-protein ligase activity	12	7.50E-06	1.02E-02
GO:0051340~regulation of ligase activity	12	1.09E-05	1.48E-02
GO:0031398~positive regulation of protein ubiquitination	12	1.55E-05	2.10E-02
GO:0031145~anaphase-promoting complex-dependent proteasomal ubiquitin-dependent protein catabolic process	11	8.84E-06	1.20E-02
GO:0051436~negative regulation of ubiquitin-protein ligase activity during mitotic cell cycle	11	8.84E-06	1.20E-02
GO:0051444~negative regulation of ubiquitin-protein ligase activity	11	1.17E-05	1.59E-02
GO:0051352~negative regulation of ligase activity	11	1.17E-05	1.59E-02
GO:0051437~positive regulation of ubiquitin-protein ligase activity during mitotic cell cycle	11	1.34E-05	1.81E-02
GO:0051439~regulation of ubiquitin-protein ligase activity during mitotic cell cycle	11	1.97E-05	2.67E-02
GO:0042274~ribosomal small subunit biogenesis	9	4.09E-11	5.60E-08
GO:0042273~ribosomal large subunit biogenesis	7	7.21E-08	9.88E-05
